# Transcriptome analysis and molecular marker discovery in *Solanum incanum* and *S. aethiopicum*, two close relatives of the common eggplant (*Solanum melongena*) with interest for breeding

**DOI:** 10.1186/s12864-016-2631-4

**Published:** 2016-04-23

**Authors:** P. Gramazio, J. Blanca, P. Ziarsolo, F. J. Herraiz, M. Plazas, J. Prohens, S. Vilanova

**Affiliations:** Instituto de Conservación y Mejora de la Agrodiversidad Valenciana, Universitat Politècnica de València, Camino de Vera 14, 46022 Valencia, Spain

**Keywords:** *Solanum incanum*, *S. aethiopicum*, Eggplant genepool, De novo transcriptome, Gene annotation, Molecular marker discovery

## Abstract

**Background:**

*Solanum incanum* is a close wild relative of *S. melongena* with high contents of bioactive phenolics and drought tolerance. *S. aethiopicum* is a cultivated African eggplant cross-compatible with *S. melongena*. Despite their great interest in *S. melongena* breeding programs, the genomic resources for these species are scarce.

**Results:**

RNA-Seq was performed with NGS from pooled RNA of young leaf, floral bud and young fruit tissues, generating more than one hundred millions raw reads per species. The transcriptomes were assembled in 83,905 unigenes for *S. incanum* and in 87,084 unigenes for *S. aethiopicum* with an average length of 696 and 722 bp, respectively. The unigenes were structurally and functionally annotated based on comparison with public databases by using bioinformatic tools. The single nucleotide variant calling analysis (SNPs and INDELs) was performed by mapping our *S. incanum* and *S. aethiopicum* reads, as well as reads from *S. melongena* and *S. torvum* available on NCBI database (National Center for Biotechnology Information), against the eggplant genome. Both intraspecific and interspecific polymorphisms were identified and subsets of molecular markers were created for all species combinations. 36 SNVs were selected for validation in the *S. incanum* and *S. aethiopicum* accessions and 96 % were correctly amplified confirming the polymorphisms. In addition, 976 and 1,278 SSRs were identified in *S. incanum* and *S. aethiopicum* transcriptomes respectively, and a set of them were validated.

**Conclusions:**

This work provides a broad insight into gene sequences and allelic variation in *S. incanum* and *S. aethiopicum*. This work is a first step toward better understanding of target genes involved in metabolic pathways relevant for eggplant breeding. The molecular markers detected in this study could be used across all the eggplant genepool, which is of interest for breeding programs as well as to perform marker-trait association and QTL analysis studies.

**Electronic supplementary material:**

The online version of this article (doi:10.1186/s12864-016-2631-4) contains supplementary material, which is available to authorized users.

## Background

The global production of eggplants has considerably increased, especially in Asia and Africa, rising from 29 to 49 millions of tons in the last decade [1]. Despite its economic importance, compared to other major vegetable crops, few efforts have been made to use related species for the genetic enhancement of common eggplant (*Solanum melongena* L.). In this respect, resistance and tolerance to biotic and abiotic stresses, as well as high levels of bioactive compounds have been found in *S. melongena* relatives [2–4], but they have not been widely used in breeding programs.

Although *S. melongena*, which was domesticated in Southeast Asia [5], is by far the economically most important cultivated eggplant, there are two other cultivated eggplant species of African origin, *S. aethiopicum* L. and *S. macrocarpon* L., which are major vegetable crops [6, 7]. The three species, together with a large number of wild species from the eggplant clade and the anguivi clade of *Solanum* subgenus Leptostemonum [8], form part of the same genepool. Most of these species can be successfully hybridized with *S. melongena* [4]. Among them the wild *S. incanum* L. and the cultivated *S. aethiopicum* have been regarded as valuable sources of variation for *S. melongena* breeding [3, 10–13].

*Solanum incanum*, which has been regarded for a time as the wild ancestor of *S. melongena* [14], is naturally distributed in desert and dryland areas in a broad area between northern Africa and the Middle East of Pakistan [15]. *S. incanum* is considered a powerful source of bioactive phenolics, mainly chlorogenic acid (5-O-caffeoyl-quinic acid) and to lesser extent N-(E)-caffeoylputrescine, 3-O-malonyl-5-O-(E)-caffeoylquinic acid and 5-O-malonyl-4-O-(E)-caffeoylquinic acid [12], showing a content several times higher than that of cultivated eggplants [12, 13, 16 ]. Moreover, *S. incanum* presents resistance at some fungal diseases, like *Fusarium oxysporum* and *Phomopsis vexans* and tolerance to abiotic stresses such as drought [2, 14, 17]. Fully fertile interspecific hybrids have been obtained between *S. incanum* and *S. melongena* with a regular meiosis [12, 14, 18, 19], as well as a backcross population to *S. melongena* that has allowed the development of an interspecific genetic linkage map [20]. *S. aethiopicum* is the second most important cultivated eggplant, and its cultivation is widespread in Africa, mainly in the west and central part, as well as in some parts of Caribbean, Brazil and south Italy [7, 21, 22]. Generally, this species is divided in four cultivar groups, namely Aculeatum, Gilo, Kumba, and Shum [23], with the Gilo group, used for its edible oval to rounded fruits, the most important group in the *S. aethiopicum* complex [3, 22, 23]. *S. aethiopicum* is of interest for *S. melongena* breeding as resistance to fungi (*Fusarium oxysporum*, *F. solani*, *Pythium vexans*, *Phytophthora parasitica*), bacteria (*Ralstonia solanacearum*), insect (*Leucinodes orbonalis*) as well as root-knot nematodes (*Meloidogyne incognita*) has been found in different materials of this species [9, 24–26]. Although different degrees of fertility have been found in interspecific hybrids between *S. melongena* and *S. aethiopicum*, backcrosses to *S. melongena* and introgression materials have been obtained [9–11]. As *S. aethiopicum* is a cultivated species, it does not present undesirable traits characteristic of wild species (e.g., prickliness, small fruit size, high content in saponins and glycoalkaloids, seed dormancy, etc.) that have to be removed in breeding programs. Also interspecific hybrids between *S. melongena* and *S. aethiopicum* are highly vigorous and of interest for being used as rootstocks of *S. melongena* [26].

Despite the importance of eggplants for security food for millions of people, genomics studies in this group have been limited. Only *S. melongena* has received some attention, with several intraspecific and interspecific genetic maps [20, 27–30], collections of molecular markers [29, 31, 32] a set of 16000 unigenes [27], a de novo transcriptome assembly [33], and a draft genome sequence [34] being available. Genomic resources in *S. melongena* relatives are generally scarce. In this respect, while for *S. melongena* there are 126,715 DNA and RNA sequences deposited in NCBI [35] nucleotide database (on September 2015), of which 100,389 correspond to ESTs (expressed sequence tag) sequences, only 68 sequences are available from *S. aethiopicum*, none of which is an ESTs. In *S. incanum*, a transcriptome assembly was released recently (GAYS 00000000.1), but it is still unpublished and no analyses have been released up to now.

The only exception concerns *S. torvum* Swartz, with 133,602 DNA and RNA sequences, of which 28,745 are ESTs. *Solanum torvum*, also known as turkey berry, is used as a vegetable and medicinal plant [36–38]. Also *S. torvum* shows resistance to many plant and soil-borne disease, such as *F. oxysporum*, *Verticillium dahliae*, *P. parasitica*, *R. solanacearum*, and *Meloidogyne* spp. [2, 39, 40]. Many efforts have been done to transfer these resistances through different biotechnological approaches, due to the high sterility of hybrids obtained via conventional crosses [41–45]. However, at present, the main use of *S. torvum* is as a rootstock for eggplant [46–48].

Yang et al. [33] sequenced simultaneously the transcriptomes of *S. torvum* and *S. melongena*, providing valuable sets of unigenes and detailed information about the two species. However, this study did not include the discovery of molecular markers, which could have been of great assistance in the breeding programs within and between each species.

The aims of the present study are building two transcriptomes from *S. incanum* and *S. aethiopicum* through the generation of ESTs using RNA-Seq, providing genomic tools in these relatives of *S. melongena*. This will be the starting point for gene discovery, splicing patterns and other post-transcriptional modifications, as well as expression levels of transcripts during development and under different conditions. Furthermore, the trimmed transcripts of *S. aethiopicum* and *S. incanum* and the transcripts of *S. melongena* and *S. torvum*, downloaded from NCBI database [35], were mapped against the eggplant genome to discover the molecular variations within and between species in order to create large subsets of markers directly applicable in breeding programs along to the eggplant genepool. All this information will contribute to the utilization of these species for *S. melongena* breeding, as well as to the enhancement of the cultivated, but neglected, *S. aethiopicum*.

## Results and discussion

### Illumina paired-end sequencing and EST assembly

The new generation of high-throughput sequencing platforms and the improved algorithms for de novo transcriptome assembly has allowed the availability of transcriptomes even in non-model organisms without a reference genome [49]. In our case, in order to build a transcriptome of *S. incanum* and *S. aethiopicum*, mixed RNA from young leaf, floral bud and young fruit, was used in order to increase the heterogeneity and diversity of the transcripts. Two different libraries were constructed, one per species, and subsequently sequenced in a HiSeq 2000 sequencer (Illumina).

A total of 105,625,594 and 114,162,500 raw reads were obtained from *S. incanum* and *S. aethiopicum* respectively (Table 1), which have been deposited in the NCBI Sequence Read Archive (Bioproject SRP063088) [35]. After the filtering and trimming process, removing adapters and low Phred quality sequences, 91,579,142 and 99,012,712 high-quality sequences were obtained for *S. incanum* and *S. aethiopicum*. The trimmed reads were assembled into transcriptomes using Trinity software [50], generating 108,322 transcripts for *S. incanum* and 106,660 for *S. aethiopicum* (Additional file 1). Subsequently, in order to test the overall assembly quality, the clean reads were mapped against the transcriptomes using Bwa [51], which is an ultrafast and memory-efficient mapper particularly good at aligning reads between 50 to 100 bp. The large number of reads properly mapped, specifically 94.3 % for *S. incanum* and 95.9 % for *S. aethiopicum*, confirmed the high quality of Trinity assembly. The total length of assembled transcripts for *S. incanum* and *S. aethiopicum* was about 102 and 92 Mbp with an average length of 946 and 868 bp, respectively. In recent years the assemblies have been improved progressively as a result of the advances in sequencing platforms, especially Illumina [52–54].

**Table 1:** Statistics of *S. incanum* and *S. aethiopicum* assembled transcripts and unigenes, using Trinity software

		*S. incanum*	*S. aethiopicum*
Raw reads		105,625,594	114,162,500
	Sequence lenght	101	101
	Mean sequence quality (Phred Score)	36.03	36.02
	%CG	43	43
High-quality reads		91,579,142	99,012,712
	Sequence lenght	70-101	70-101
	Mean sequence quality (Phred Score)	36.88	36.87
	%CG	42	42
Transcript		108,322	106,660
	Max lenght	12,202	12,179
	Avarage lenght	946	868
	N50	1,693	1,455
	Total residues	102,496,435	92,629,886
Unigenes		83,905	87,084
	Max lenght	12,181	12,159
	Avarage lenght	696	722
	N50	1,153	1,139
	Total residues	58,447,674	62,899,378

Data correspond to the results of RNA-Seq projects by HiSeq 2000 sequencer (Illumina) of total RNA from three tissues of *S. incanum* and *S. aethiopicum* and subsequent processing

Trinity software determines splice variants (isoforms) and distinguishes transcripts from recently duplicated and identified allelic variants [50]. To obtain a set of single-copy gene locus (unigene), only the most expressed transcript from the isoforms of each locus was chosen, using the RSEM software (RNA-Seq by Expectation-Maximization) [55]. A total of 83,905 unigenes were identified in *S. incanum* and 87,084 in *S. aethiopicum*, showing that 22.5 % and 18.3 % of transcripts were splice variants respectively (see Additional file 2).

**Fig. 1:**
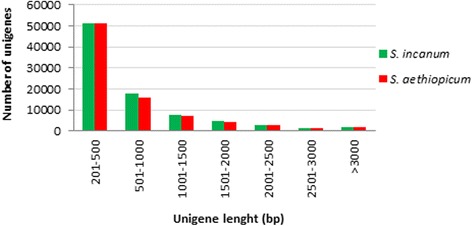
Length distribution (bp) of *S. incanum* and *S. aethiopicum* unigenes

**Fig. 2:**
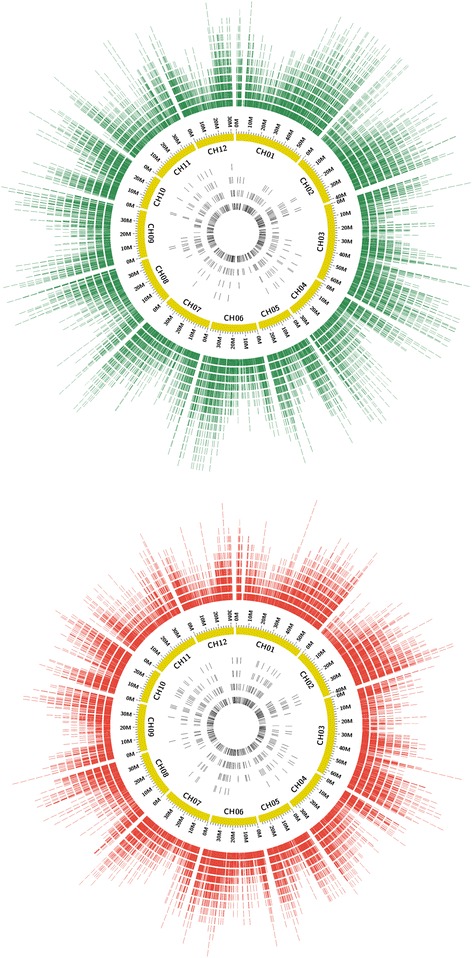
Distribution of *S. incanum* (*top*) and *S. aethiopicum* (*bottom*) assembled unigenes and SSRs on *S. melongena* genome. In the outer histogram, depicted in *green* for *S. incanum* and in *red* for *S. aethiopicum*, are represented the assembled unigenes along the eggplant genome (*yellow* ring). Only unigenes which have given an E-value 0.0 via BlastN search were shown. In the inner histograms, in *black*, the distribution of the SSRs detected in each species is represented

Even though the 60 % of unigenes had between 201 and 500 bp, more than 20 % of them were longer than 1 kbp. The length distribution of unigenes is shown in Fig. 1. In order to obtain the physical position of the unigenes, a BlastN against the *S. melongena* genome was performed and the results are illustrated in the Circos plot in Fig. 2 and Additional file 3 [56]. The distribution of unigenes was uneven along the eggplant genome. Most unigenes clustered in areas which could correspond to the short and long arms of chromosomes, while a lower unigene density was observed in regions which could correspond to the centromere and pericentromeric regions, based on the observation of Doganlar et al. [28]. This gene distribution is similar to the observed in other species [57, 58]. Our sets of unigenes are much higher than those obtained for *S. melongena* and *S. torvum. S. melongena* unigenes set consisted of 16,245 [59] and 34,174 unigenes [33], while in *Solanum torvum* 38,185 unigenes were obtained [33]. A deeper sequencing and better coverage were probably the reasons of the greatest number of our *S. incanum* and *S. aethiopicum* datasets unigenes. In fact the number of our total residues was 58,447,674 for *S. incanum* and 62,899,378 for *S. aethiopicum*, higher than those obtained by Fukuoka et al. [59] and Yang et al. [33] in *S. melongena* (50,438,137 and 27,771,410, respectively), and Yang et al. [33] in *S. torvum* (30,868,727). These higher numbers of unigenes has been observed in other plant transcriptomes recently published [60–62] in which the number of raw reads obtained was quite large.

### Annotation of *S. incanum* and *S. aethiopicum* transcriptomes

Transcriptome annotation provides insight into the structural, functions and biological processes of assembled unigenes [63]. The functional annotation was performed using the assembled unigenes as query in BlastX searches against the three major protein databases and tomato protein database in this sequential order: Swiss-Prot [64], ITAG2.4 [65], Arabidopsis proteins [66] and Uniref90 [67]. Moreover a BlastX analysis was performed against the non-redundant (NR) protein database of NCBI [35] and the GO terms (Gene Ontology) and EC number (Enzyme Commission) have been assigned through the Blast2GO software [68].

A total of 30,630 (36.5 %) *S. incanum* and 34,231 (39.3 %) *S. aethiopicum* have shown at least one hit in the protein databases. Most of the unigenes (57.5 % for *S. incanum* and 57.3 % for *S. aethiopicum*) were annotated using the manually reviewed Swiss-Prot database and ITAG 2.4 (34.9 % and 33.2 %) and less using Uniref90 (7.3 % and 9.3 %) and Arabidopsis protein database (0.3 % and 0.2). The unigenes annotated under different protein database are reported in Additional file 4.

Even though the percentage of unigenes annotated in the protein databases seems to be quite low, the total number, 30,630 for *S. incanum* and 34,231 for *S. aethiopicum*, is consistent with the number of protein-coding genes described in tomato (34,727) [69] and in previous works in other plant species. For instance in pepper transcriptome (*Capsicum annum* L.) [70] 24,003 out of 31,196 unigenes were annotated in protein databases as well as 32,410 out of 68,132 unigenes in *Oryza officinalis* Wall. ex Watt [71] and 34,368 out of 82,036 unigenes in litchi (*Litchi chinesis* Sonn.) [72]. Similarly, in *S. melongena* and *S. torvum* 28,016 and 29,845 unigenes were annotated, respectively [33].

A large portion of hitless unigenes were short sequences between 200 and 500 bp. This huge set of non-annotated short sequences has been observed in recent published transcriptomes, in which large amount of raw reads have been obtained [61, 73]. Besides 3′ or 5′ untranslated regions (UTRs) and intron sequences from non-mature mRNAs, several authors have described that some of these sequences could be noncoding RNAs (ncRNAs) [74]. Unfortunately at the present the ncRNA is still in their early stages and just some mammalian entries were uploaded to IncRNA database [75]. Up to now, the only plant that has received some attention is *Arabidopsis thaliana* where 13,000 RNAs were found transcribed from intergenic regions [76, 77].

Gene ontology provides a systematic language in three key biological domains shared by all organisms: molecular function, biological process and cellular component to unify the representation of gene features across all species [39]. GO terms are structured as a graph and can be distributed in levels. Level 1 represents the most general categories and provides the most coverage, whereas higher levels provide more specific information and less coverage [39]. Briefly, level 1 is a general description of a process whereas higher levels provide a more specific description.

**Fig. 3:**
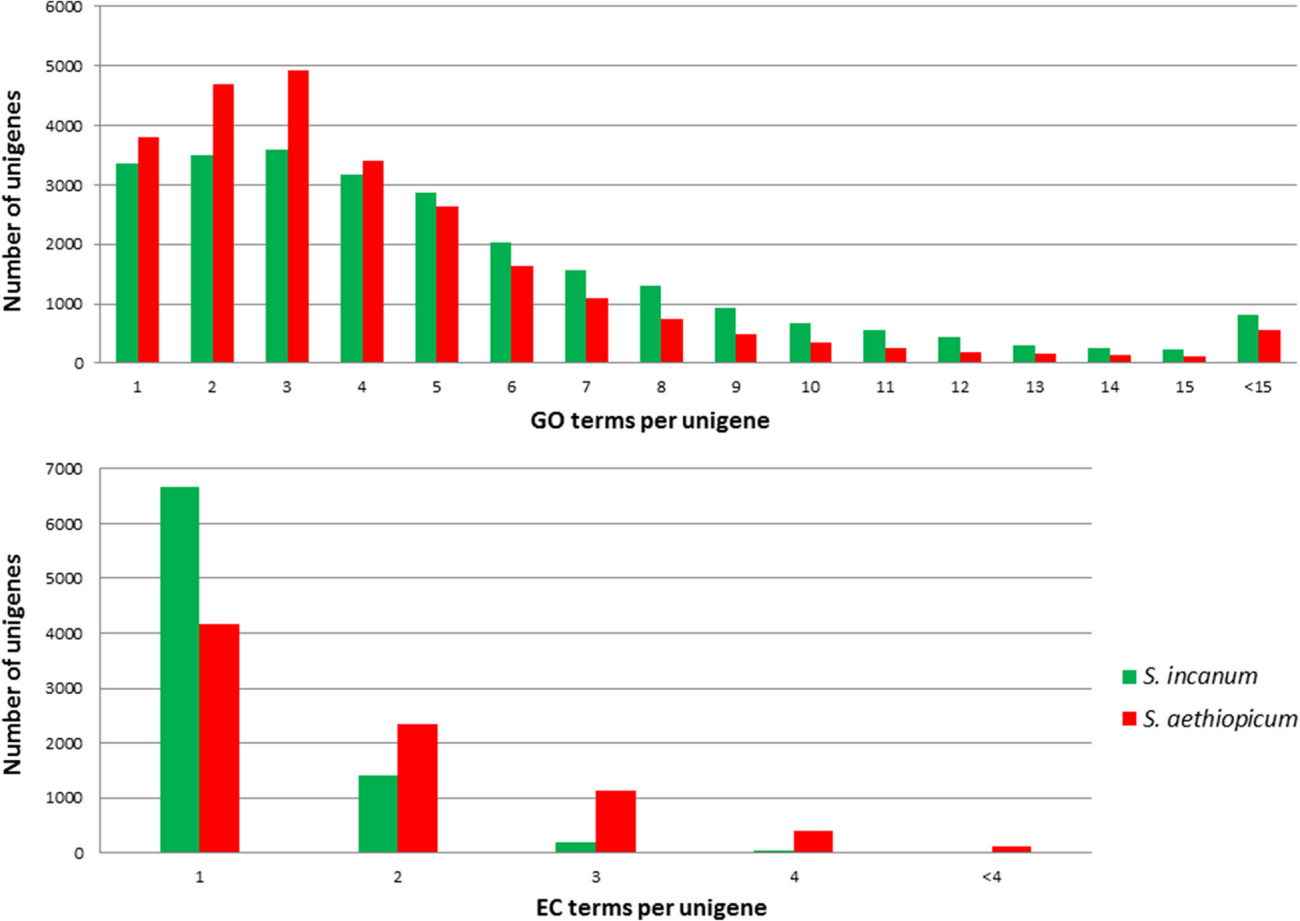
Number of GO terms per unigene (*top*) and number of EC terms per unigene (*bottom*) distribution in *S. incanum* and *S. aethiopicum* transcriptomes

A total of 136,904 and 109,044 GO terms were assigned to 25,650 (30.5 %) and 25,169 (28.9 %) unigenes in *S. incanum* and *S. aethiopicum* respectively. The GO annotation results are presented in Additional file 5. The GO terms per unigenes ranged from 1 to 92 for *S. incanum* and from 1 to 55 for *S. aethiopicum*, although most of the unigenes have 1 to 10 GO terms (Fig. 3). The unigenes were also annotated with EC number [78], which identifies the reactions they catalyze. EC numbers were assigned to 8,343 (9.9 %) and 14,524 (16.6 %) unigenes, varying from 1 to 9 per unigene, although almost 80 % presented only one EC number (Fig. 3).

**Fig. 4:**
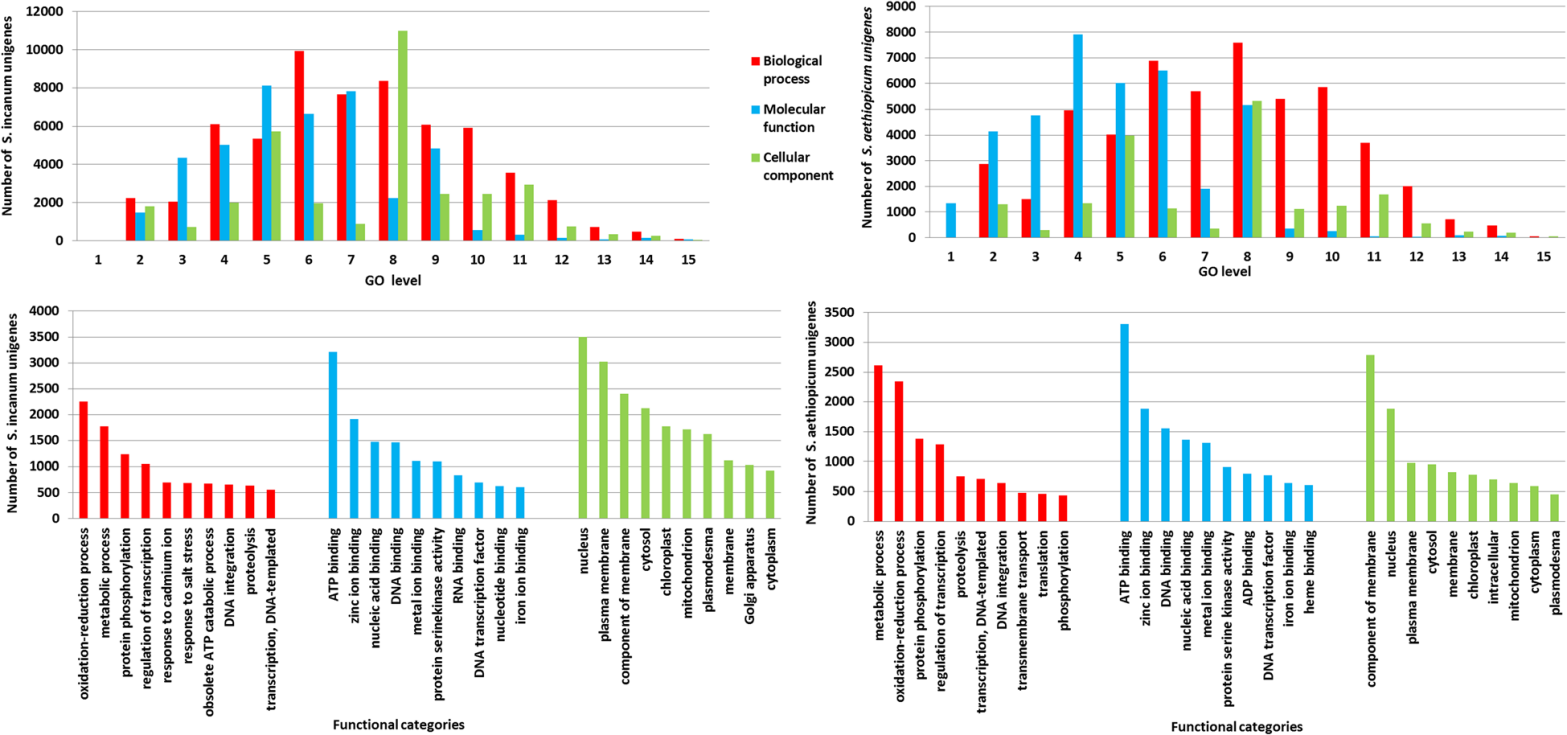
GO level distribution (*top*) and functional categories annotation (*bottom*) of the *S. incanum* (*left*) and *S.aethiopicum* (*right*) unigenes. On the *top* are shown the distributions of GO level in the following functional categories: biological process, molecular function and cellular component. On the *bottom* are represented the descriptions of the most abundant gene products for each functional category of GO ontologies: biological process, molecular function and cellular component

The majority of GO terms (44.6 % for *S. incanum* and 47.4 % for *S. aethiopicum*) were related to biological processes (Fig. 4). Most of them had a GO annotation level in the range of 4 to 10. Biological processes such as oxidation-reduction and metabolic processes, protein phosphorylation and regulation of transcription are usually specific of tissues in a developmental stage [79]. Molecular functions have been assigned to 30.7 % and 35.4 % of ontologies, most of them showing a GO annotation level of 3 to 9 and being the binding activities the most represented. The remaining 24.7 % and 17.3 % of annotated unigenes have shown a cellular component GO term, mostly related to nucleus, plasma membrane, cytosol, as well as chloroplast and mitochondria. The distribution of GO level for this category is quite uniform, with the exceptions of levels 5 and 8.

The Kyoto Encyclopedia of Genes and Genomes (KEGG) is an integrated database resource, which links genomic data with functional information to standardize gene annotation [80]. Using Blast2GO software the annotated unigenes were blasted against the KEGG pathway database in order to dissect the molecular interaction among them. A total of 11,151 (13.2 %) *S. incanum* unigenes were assigned to 146 KEGG biological pathways, involving 378 enzyme types and 879 EC numbers. Regarding *S. aethiopicum* 13,101 unigenes (15.0 %) were ascribed to 147 KEGG pathways, including 356 different enzymes and 821 EC numbers. In *S. incanum* the three most enriched pathway were the biosynthesis of antibiotics, which included 713 unigenes (map01130), made mainly by phosphohexokinase (21 unigenes), dehydrogenase (18) and dehydrogenase (NAD+) (18) enzymes, Purine metabolism pathway (567 unigenes, map00230) was mostly represented by phosphatase (194 unigenes), adenylpyrophosphatase (130) and RNA polymerase (79) enzymes, and starch and sucrose metabolism pathway (516 unigenes, map map00500) was composed especially by pectin demethoxylase (85 unigenes), pectin depolymerase (64) and UDP synthase (54) enzymes. In *S. aethiopicum* the most enriched pathway was purine metabolism (map00230), which encompasses 1404 unigenes, mostly constituted by phosphatase (1160 unigenes), adenylpyrophosphatase (630) and RNA polymerase enzymes (92), followed by thiamine metabolism pathway (1172, map00730), made basically for phosphatase enzymes (1160 unigenes), and biosynthesis of antibiotics pathway (639 unigenes, map01130) including 168 enzyme types of which the most represented were dehydrogenase (20 unigenes), phosphohexokinase (18) and transaminase (16) enzymes. The biological pathways maps are reported in the Additional file 6 and the KEGG annotation results are compiled in the Additional file 7.

In order to establish a set of orthologs and gene model prediction, a best reciprocal Blast hits was performed with the tomato reference genome (version SL2.50) [69]. Up to now, the closest phylogenetically published genome of *S. incanum* and *S. aethiopicum* is the one of *S. melongena* [33]. Even though this first version has provided valuable information for eggplant breeding programs, it is just a draft, requiring a deeper and most complete sequencing. On the other hand, the *S. lycopersicum* genome is the most complete and accurate in genus *Solanum*. In the last version, SL2.50, the tomato genome scaffolds were re-ordered and re-oriented, and the gap sizes between scaffolds were set using FISH (Fluorescence in situ hybridization) and optical mapping. For that reason, apart from *S. melongena*, a BlastN was performed against *S. lycopersicum*. In the Additional file 8 the Blast hits results between the assembled unigenes and *S. lycopersicum* genome version SL2.50 are reported. A total of 16,388 (19.5 %) and 17,630 (20.2 %) unigenes have presented orthologs with tomato reference genome in *S. incanum* and *S. aethiopicum* respectively (Additional file 9), while between the two transcriptomes 46,498 orthologs were found.

Regarding structural annotation, the ORF (Open reading frame) detection was performed using ESTScan software [81], which predicted 35,943 ORFs (42.8 % of the total unigenes residues; Additional file 10) in *S. incanum*. The total number of nucleotides in S. *incanum* ORFs was 39,611,611 (67,7 % of total *S. incanum* unigenes assembled residues). In *S. aethiopicum* 40,353 ORFs were predicted (46.3 % of the total unigenes), which are constituted by 43,653,585 nucleotides (69.4 % of total *S. aethiopicum* unigenes assembled residues Additional file 10). Furthermore using est2genome [82] the intron regions were detected, providing valuable information about gene structure as well as in the task of primers design, avoiding regions in the intron edges proximity. In 12,368 *S. incanum* unigenes (14.7 % of the total unigenes) 59,501 introns were predicted, while 65,996 introns were detected in 13,661 *S. aethiopicum* unigenes (15.6 %) (Additional file 10). Both species have exhibited an average of 4.8 introns per unigene with a maximum of 49 for *S. incanum* and 56 for *S. aethiopicum*. The low percentage of introns detected in the unigenes is probably due to that the 60 % of unigenes had between 201 and 500 bp and for their small size did not contain any intron.

### Molecular markers discovery and validation

#### Single nucleotide variations (SNVs)

During the last decade Next-Generation Sequencing (NGS) techniques have allowed the development of large molecular marker collections with modest investments even in non-model species [83, 84]. These collections enable the location of thousands of single polymorphisms along the genome as well as the development of high-density genetic maps, arrays and genotyping assays [85–87].

Although genomic resources have been developed in eggplant (e.g., Barchi et al., [29]; Yang et al., [33] Hirakawa et al., [34]), few genomic information is available for closely related species. In the present study, large subsets of SNPs (Single Nucleotide Polymorphisms) and INDELs (insertion/deletions) have been identified to assist efficiently plant breeding projects and diversity studies. The SNP calling was performed for the species sequenced in the present study (*S. incanum* and *S. aethiopicum*) and for the two other species of the eggplant genepool (*S. melongena* and *S. torvum*), whose transcriptomes have already been sequenced [33]. The reads of the four transcriptomes have been mapped against the eggplant genome and SNPs detected using Freebayes SNP caller [88], as detailed in Methods section. The complete information of SNP calling is provided in Additional file 11. A set of 36 SNVs (Single nucleotide variations), three per eggplant chromosome, were selected for validation in the *S. incanum* and *S. aethiopicum* accessions using the HRM (High Resolution Melting) technique [89]. Of these, a total of 96 % were correctly amplified and in all of them polymorphisms detected have been confirmed (see Additional file 12).

For each of the four species, the intraspecific and interspecific polymorphisms were identified by filtering the VCF (Variant Call Format) file through the species ID number. In addition the INDELs were separated from the SNPs applying the VKS filter (it is not a SNP). The results of SNP calling are reported in Table 2. *S. aethiopicum* presented the highest value of intraspecific SNVs, with 159,571 SNPs and 4,556 INDELs. Many less intraspecific polymorphisms (12,396, of which 11,861 were SNPs and 535 INDELs) were identified in *S. incanum*. This suggests that the *S. aethiopicum* accession used presented a larger degree of heterozygosity than the S. incanum accession. Finally, in *S. melongena* and *S. torvum* [33] 2780 SNVs (2660 SNPs and 120 INDELs) and 25,147 SNVs (18,829 SNPs and 6,318 INDELs) intraspecific polymorphisms were discovered respectively. The high level of intraspecific variation in *S. aethiopicum*, *S. torvum* and *S. incanum* in comparison with *S. melongena* are probably due to the higher degree of autogamy and breeding selection for uniformity of the latter [90].

**Table 2:** Single nucleotide variations statistics for the *S. incanum* and *S. aethiopicum* transcriptomes

SNVs intraspecific variations			INDELs	SNPs	Total SNVs
	*S. incanum*		535	11,861	12,396
		filtered	28	385	
	*S. aethiopicum*		4,556	159,571	164,127
		filtered	312	5,804	
	*S. melongena*		120	2,660	2,780
		filtered	6	57	
	*S. torvum*		6,318	18,829	25,147
		filtered	26	90	
SNVs interspecific variations			INDELs	SNPs	Total SNVs
	*S. incanum* and *S. aethiopicum*		586	14,576	15,162
		filtered	29	649	
	*S. incanum* and *S. melongena*		3,673	102,104	105,777
		filtered	253	4,184	
	*S. incanum* and *S. torvum*		96,799	491,965	588,764
		filtered	760	3,995	
	*S. aethiopicum* and *S. melongena*		2,392	73,059	75,451
		filtered	165	3,277	
	*S. aethiopicum* and *S. torvum*		83,106	420,685	503,791
		filtered	604	3,229	
	*S. melongena* and S. *torvum*		92,323	464,071	556,394
		filtered	722	3,682	
	*S. incanum*, *S. aethiopicum* and *S. melongena*		108	41	149
		filtered	8	2	
	*S. incanum*, *S. aethiopicum* and *S. torvum*		908	198	1,106
		filtered	10	3	
	*S. incanum*, *S. melongena* and *S. torvum*		8,022	1,265	9,287
		filtered	118	15	
	*S. aethiopicum*, *S. melongena* and *S. torvum*		6,986	886	7,872
		filtered	102	12	
	*S. incanum*, *S. aethiopicum*, *S. melongena* and *S. torvum*		50	0	50
		filtered	2	0	

SNVs (SNPs and INDELs) have been identified by the Freebayes SNP caller. The interspecific and intraspecific variations, as well as all species combination, have been determined by filtering ID (identification) species number. Every species and species comparison present an unambiguous identification number. The INDELs have been separated from SNPs applying the VKF filter to the total SNVs. The filtered SNPs and INDELs have been obtained by adding the CS60, HV0.05, CL60 and CEF filters

**Fig. 5:**
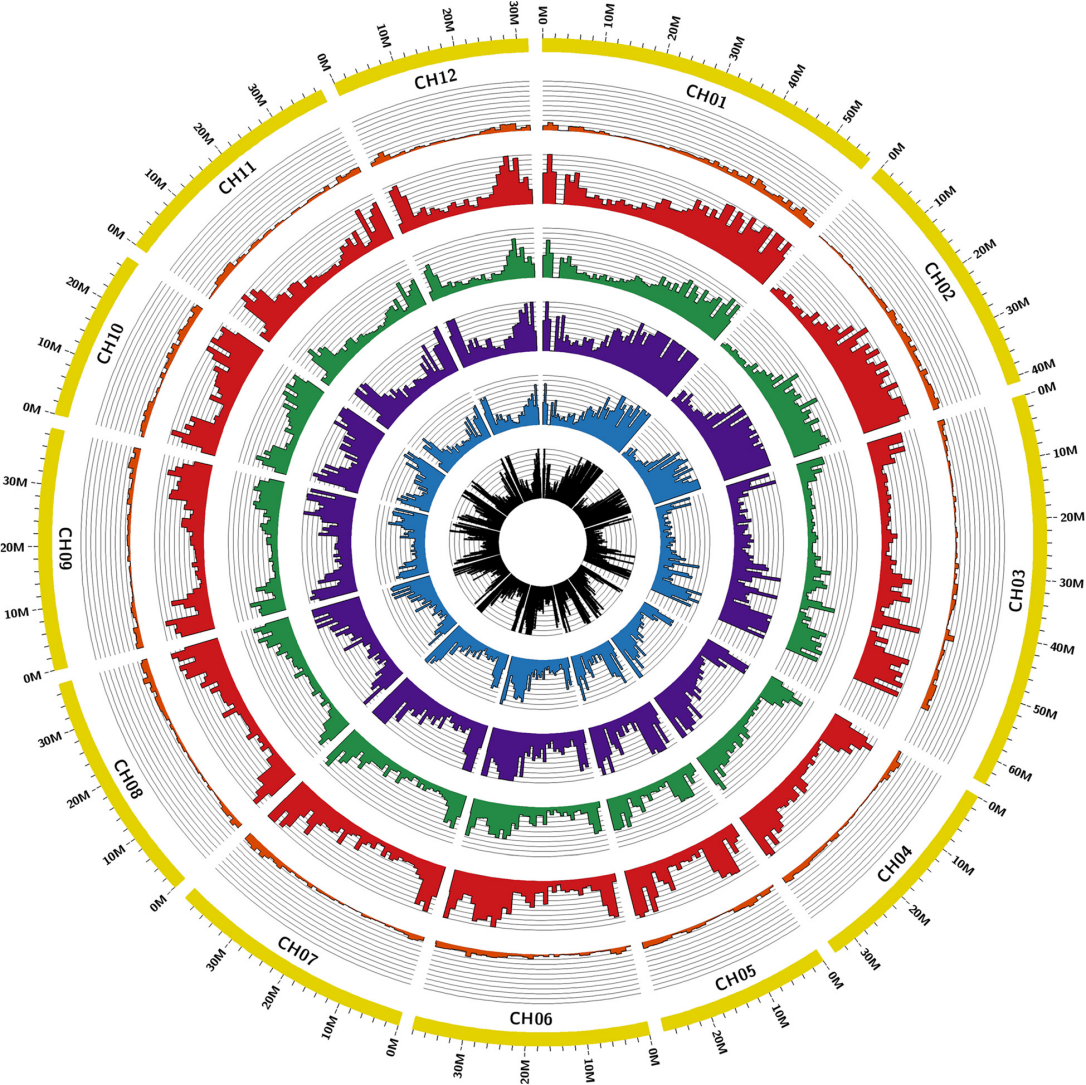
Distribution of interspecific SNVs graphically depicted on eggplant genome. The histogram distributions, represented in different colors, illustrate the interspecific variations, two species at a time, along the eggplant genome (outer circle, yellow ring). From outer to inner are shown: *S. incanum* vs *S. aethiopicum* comparison (orange histogram), *S. incanum* vs *S. melongena* comparison (red histogram), *S. aethiopicum* vs *S. melongena* comparison (green histogram), *S. incanum* vs *S. torvum* comparison

The interspecific SNVs were detected in the comparisons between two species or three species at the same time. An interspecific SNV were selected when the species were homozygous for a specific allele, but different allele from one species to another (Table 2). The Circos plot in Fig. 5 shows the six combinations of interspecific SNVs comparison and the location of the variations in the eggplant genome. As in the case of unigenes distribution along the S. melongena genome, the SNVs are less represented in areas which could correspond to centromere and more represented in regions which could correspond to the chromosome arms. *Solanum torvum* presented the greater number of interspecific SNVs. A total of 1,648,949 polymorphisms (1,376,721 SNPs and 272,228 INDELs) have been detected in *S. torvum*, 588,764 of which are with *S. incanum*, 556,394 with *S. melongena* and 503,791 with *S. aethiopicum*. Out of the four species, *S. torvum* is the most phylogenetically distant [8]; this explains the large number of interspecific polymorphisms. *Solanum melongena* presented the second most abundant set of interspecific polymorphisms, 737,622 SNVs (639,234 SNPs and 98,388 INDELs). The comparison with *S. aethiopicum* detected 75,451 SNVs and 105,777 with *S. incanum*.

*S. melongena* and *S. incanum*, being members of the eggplant clade [8] we would expect less polymorphisms between these two species than between *S. melongena* and *S. aethiopicum*, which belongs to the anguivi clade. This discrepancy could be explained by the high amount of *S. aethiopicum* intraspecific polymorphisms, which reduces the differences in the number of SNVs between *S. melongena* and *S. aethiopicum*. Finally the lowest variation was found between *S. incanum* and *S. aethiopicum* (15,162 SNVs). The interspecific SNVs were substantially less abundant when three species were compared. The majority of polymorphisms were INDELs rather than SNPs, due to the preferential bi-allelic nature of the latter [91, 92]. The collections of variations have been larger in the combinations which included *S. melongena* and *S. torvum*, and smaller with *S. incanum* and *S. aethiopicum*. In addition 50 INDELs were detected when the four species were compared all together.

Subsequently, all intraspecific and interspecific SNVs detected in the four species were filtered in order to create subsets of the most suitable and effective variations for genotyping assays, both manually and with high throughput platforms, such as High Resolution Melting and GoldenGate Assay [89, 93, 94]. These variations have well-spaced positions, distanced more than 60 bp from another polymorphism and from the edges of the assembled transcripts. Furthermore, they could be detected by digestion with commonly used and cheap restriction enzymes.

### SSRs

The transcriptomes were examined to discover SSRs (Simple Sequence Repeat) made of di-, tri-, tetra-nucleotide motifs by using the Sputnik software [95]. In *S. incanum*, a set of 976 SSRs were identified in 954 unigenes; i.e., 1.1 % of the unigenes contained at least one microsatellite, while in *S. aethiopicum* 1,708 SSRs were detected in 1628 unigenes (1.8 %). The total number of SSRs yielded was lower than obtained in other studies [96, 97], probably due to the stringent criteria used to obtain high confident markers. The microsatellites identified are summarized in Table 3, while their location on eggplant genome is shown in Fig. 2.

**Table 3:** SSRs statistics corresponding to the *S. incanum* and *S. aethiopicum* transcriptomes

		*S. incanum*			*S. aethiopicum*		
SSRs repeat motifs		SSRs	%	Unigenes	SSRs	%	Unigenes
Dinucleotide		260	26.6	258	362	29.0	342
	AG/CT	160	61.5		187	51.7	
	AT/TA	63	24.2		98	27.1	
	AC/GT	36	13.8		76	20.9	
	CG/GC	1	0.3		1	0.3	
Trinucleotide		621	63.6	609	776	62.2	755
	AAG/CTT	169	27.2		263	33.8	
	AAC/GTT	145	23.3		173	22.2	
	AAT/ATT	82	13.2		106	13.7	
	ATC/GAT	73	11.7		63	8.1	
	AGG/CCT	53	8.5		54	7.0	
	ACC/GGT	39	6.2		41	5.3	
	AGC/GCT	34	5.4		40	5.2	
	CCG/CGG	12	1.9		17	2.2	
	ACT/AGT	10	1.6		10	1.3	
	ACG/CGT	4	0.6		9	1.2	
Tetranucleotide		95	9.8	93	110	8.8	110
	AAAG/CTTT	29	30.5		36	32.7	
	AAAT/TTTA	23	24.2		28	25.5	
	AAAC/GTTT	10	10.5		5	4.5	
	ACAT/ATGT	5	5.2		9	8.2	
	AAGG/CCTT	4	4.2		3	2.8	
	ATCC/GGAT	4	4.2		1	0.9	
	AACC/GGTT	3	3.1		4	3.6	
	AATG/CATT	3	3.1		2	1.8	
	AATT/AATT	3	3.1		6	5.5	
	AGGG/CCCT	3	3.1		1	0.9	
	Others motifs	8	8.4		15	13.6	
Total		976		954	1,248		1,270

Di-, tri- and tetranucleotide repeats and motifs identified in the *S. incanum* and *S. aethiopicum* assembled unigenes are indicated

The range of SSRs length varied between 16 and 72 nucleotides in *S. incanum* and between 16 and 74 in *S. aethiopicum* with an average value of 21 and 24 nucleotides respectively. The most represented SSRs motifs in both species corresponded to AG, AAG and AAAG in agreement to the observation in other crops [98–100]. Trinucleotide repeat motifs were the most abundant (63.6 % and 62.7 %), followed by dinucleotide (26.6 % and 28.4 %) and tetranucleotide (9.8 % and 8.9 %) repeats. The prevalence of trinucleotide motifs is well documented in literature in eggplant [29, 101] as well as in other crops [102, 103]. Metzgar et al. [104] hypothesized that non-triplet SSRs show higher risks of frameshift mutation in coding regions and the selection against these mutations would reduce their chances of fixation. Otherwise the selection against frameshift events does not occur in SSRs with a repeat length divisible by three (tri- and hexanucleotide repeats). Depending on their position in the gene, SSRs can be involved in different processes. The genic SSRs in 5′-UTR are implicated in gene transcription and gene translation while in 3′-UTR are implied in gene silencing and transcription slippage. In introns, SSRs can activate and inactivate genes [105]. The analysis of localization revealed that most of SSRs were located in ORFs, 33.5 % for *S. incanum* and 32.7 % *for S. aethiopicum*, and less in the UTRs (Table 4). In ORFs the trinucleotides repeats were the most abundant (88.7 % for *S. incanum* and 87.7 for *S. aethiopicum*), ensuring the conservation of coding capacity and better protection against big changes in frameshift which might cause dramatic effects. On the other hand dinucleotides and tetranucleotides were more abundant in the UTRs, showing no great differences between the 5′ and 3′. These results are consistent with previous study in other species [102, 106].

**Table 4:** SSRs localization in the *S. incanum* and *S. aethiopicum* transcriptomes

*S. incanum*		di-SSRs		tri-SSRs		tetra-SSRs		Total	
		N°	%	N°	%	N°	%	N°	%
	5′-UTR	63	42.2	66	44.3	20	13.5	149	15.2
	ORF	25	7.6	291	88.7	11	3.7	327	33.5
	3′-UTR	40	28.8	83	59.7	16	11.5	139	14.2
	Other	132	36.5	181	50.1	48	13.4	361	37.1
	Total	260	26.6	621	63.6	95	9.8	976	100
*S. aethiopicum*		di-SSRs		tri-SSRs		tetra-SSRs		Total	
		N°	%	N°	%	N°	%	N°	%
	5′-UTR	74	43.0	75	43.6	23	13.4	172	13.8
	ORF	29	7.1	358	87.8	21	5.1	408	32.7
	3′-UTR	73	38.0	106	52.3	13	6.7	192	15.3
	Other	186	39.0	237	49.8	53	11.2	476	38.2
	Total	362	29.0	776	62.2	110	8.8	1,248	100

The SSRs detected in the transcriptomes were checked for their position in the unigenes (ORF, 5′UTR and 3′UTR) according to the nucleotide repeats. When no precise position was established the SSRs were defined as “Other”

One polymorphic SSR was selected per chromosome to be validated in *S. incanum* and *S. aethiopicum* accessions used for the transcriptome sequencing. Eleven out of the 12 markers were correctly amplified and resulting polymorphic between the two species (Additional file 12). This indicates that the SSRs discovered in this study will provide a valuable set of molecular markers to disclose the intraspecific and interspecific variability across the eggplant genepool. The high rate of SNPs and SSRs correctly amplified is an indirect evaluation of good transcriptome assembly.

Genic SSRs present some advantages in comparison with genomic DNA-based markers. For instance, their development from RNA-Seq projects is low-cost [107]. Genic SSRs are in many cases functional markers, when changes in allelic repeats affect functions and the phenotype. Functional markers permit a direct allele selection, if there is a clear association for a target trait. Furthermore, the flanking sequences of genic SSRs are more evolutionary conserved than genomic SSRs [108, 109], allowing considerable cross-species transferability. On the basis of its properties, the uses of genic SSRs are multiple, allowing genome and comparative mapping, genetic diversity analysis, QTL studies, gene tagging, association mapping and functional genomics [110, 111].

## Conclusions

In this study we present a de novo assembly and analysis of *S. incanum* and *S. aethiopicum* transcriptomes obtained by RNA-Seq. As a result of the annotation of these two common eggplant relatives, a broad overview of expressed genes was obtained. The annotation of the two transcriptomes has provided valuable information on function and structure of the assembled unigenes, which will allow the detection of candidate genes for important breeding traits in eggplant genepool. The large amount of intraspecific and interspecific molecular markers, genic SSRs and SNVs, identified in our transcriptomes and in the *S. melongena* and *S. torvum* transcriptomes [33], will be extremely helpful for the breeding programs, although a deeper comparison between the four transcriptome it would be of great interest. In particular filtered SNVs will allow accurate genotyping assays through high throughput platforms or arrays increasing the efficiency and rapidity of the programs. Overall, the information produced in this study provides a valuable genomic resource in two non-model species, opening the door to further studies as gene tagging, comparative mapping, association mapping for enhancing eggplant genomics and breeding.

## Methods

### Plant material

**Fig. 6:**
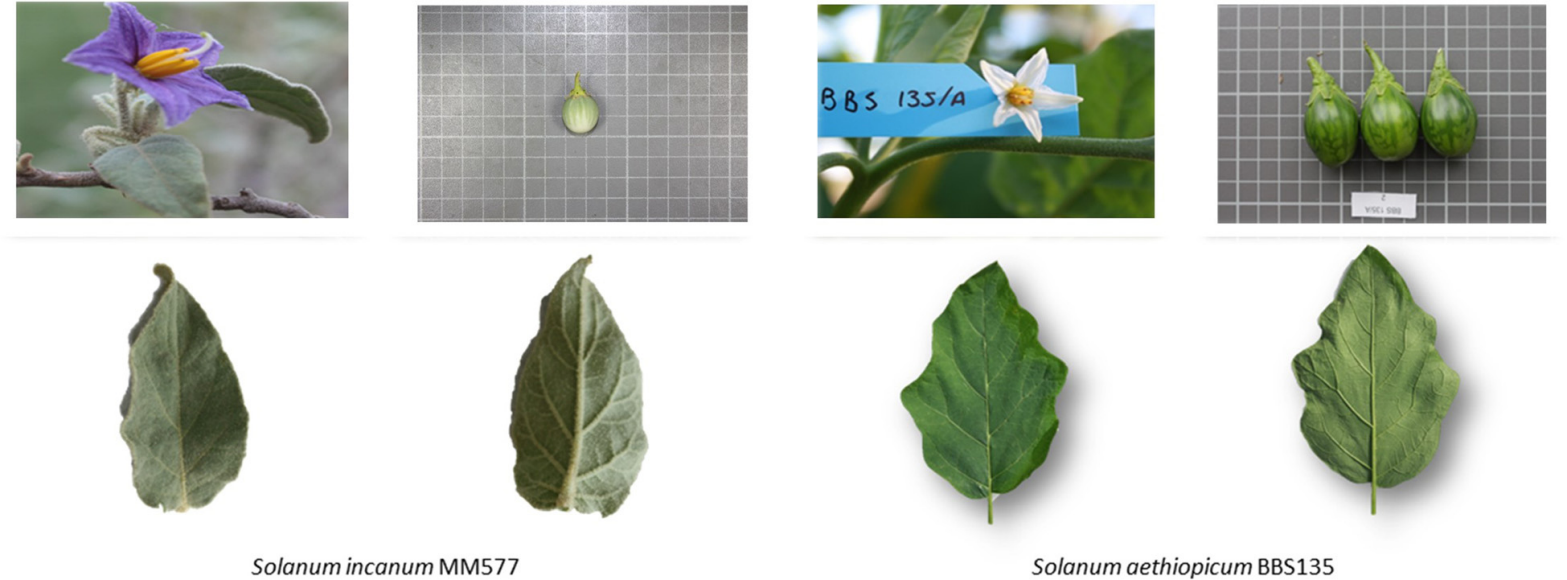
Flower, fruit and leaf of *S. incanum* (*left*) and *S. aethiopicum* (*right*). The cells in the fruit picture grid have a size of 1 cm × 1 cm

The materials used were *S. incanum* accession MM577, which was collected in the wild in Israel and *S. aethiopicum* accession BBS135, which belongs to the Gilo cultivar group and was originally collected in Ivory Coast. *Solanum incanum* accession MM577 is a spiny plant with small green rounded fruit and purple corolla while the *S. aethiopicum* accession BBS135 is thornless and presents green obovoid fruits and white corolla (Fig. 6). *Solanum incanum* accession MM577 has been used by our group as a parental to develop an interspecific genetic linkage map with *Solanum melongena* in which the candidate genes involved in the core chlorogenic acid synthesis pathway were mapped [20].

Plants of both accessions were grown in a greenhouse at the Universitat Politècnica de València (Valencia, Spain). Tissue samples were taken all at once at the stages of young leaf, floral bud and young fruit from a single plant and immediately frozen in liquid nitrogen and stored at-80 °C until used for RNA extraction.

### RNA extraction for Illumina sequencing

About 100 mg of tissue were powdered in liquid nitrogen with a mortar and pestle. TRI Reagent® Protocol (Sigma-Aldrich, St. Louis, USA) was used for the total RNA extraction. In order to avoid DNA contamination DNase I Recombinant, RNase-free (Roche, Basel, Switzerland) was used. RNA integrity was confirmed by agarose electrophoresis and RNA quantification was performed using a Nanodrop ND-1000 spectrophotometer (Thermo Scientific, Wilmington, USA). Equal amounts of total RNA from each tissue were pooled for each accession and send to Macrogen Korea (Seoul, South Korea). After the construction of paired-end library (insert size of 300 bp), RNA-Seq was performed in HiSeq 2000 sequencer (Illumina, San Diego, USA). The raw sequences obtained are available in the Sequence Read Archive at NCBI [35] at the accession number (SRS1054263) for *S. incanum* and at the accession number (SRS1052489) for *S. aethiopicum*.

### Sequence data analysis and De novo assembly

The quality of the reads generated by Illumina was checked using the FastQC program [112]. In order to obtain high-quality data, the raw reads were pre-processed and trimmed using in-house developed software, NGS_CRUMBS [113]. Through the different utilities the adapters used during the sequencing process were removed, as well as, low quality sequences, with a Phred quality score Q < 20 and ambiguous sequences with N. The trimmed reads were finally assembled into transcripts using Trinity [50], using default setting, which was specifically developed for de novo transcriptome assembly and for short-read RNA-Seq like Illumina HiSeq 2000.

In order to reduce the redundancy, the assembled transcriptomes were screened with CAP3 program [114]. CAP3 uses base quality values, merging transcripts which overlap at least 200 bp with an identity of 99 %. After that, to remove low complex sequences, the transcripts which have shown a DUST score less than 7 were masked [115]. The estimation of transcript expression levels were calculated using RSEM software [55] and subsequently the most expressed transcripts of each Trinity transcript cluster were selected to create a set of unigenes for each species. Then both sets of unigenes were blasted (cut-off value of 1e-20) against the eggplant genome in order to obtain the physical position. The distribution of unigenes over eggplant genome was graphically depicted with Circos software [56].

### Structural and functional annotation

The set of assembled transcripts was compared using BlastX (cut-off value of 1e-20) against four public protein databases in the following order: Swiss-Prot [64], ITAG2.4 [65], Arabidopsis [66] and UniRef90 [67]. If a transcript gave a blast hit in the first database, no further searches were done, otherwise a second, third or fourth blast was performed.

Subsequently, a functional annotation was realized using Blast2GO software [68] to assign at the transcripts the corresponding GO terms [116] and EC number [78]. For this purpose a BlastX (cut-off value of 1e-20) was performed in the NR database [35] and the resulting hits were mapped into gene ontology database to assign the correspondent annotation. Blast2GO was used also to obtain the KEGGs pathways from the Kyoto Encyclopedia of Genes and Genomes database (version 73.0, January 1, 2015) [80].

Additionally, best reciprocal hits with BlastN (cut-off value of 1e-20) were performed with tomato genome (version SL2.50) [69] to detect orthologs. On the other hand the tomato genome was employed to predict gene model and intron frames, using est2genome software [82]. ORFs were predicted with ESTScan program [81].

### Mapping transcriptomes against eggplant genome

The high-quality clean reads from our RNA-Seq experiment (*S. incanum* and *S. aethiopicum*) were aligned against the eggplant genome using the Top Hat program [117]. The TopHat pipeline is very fast and specifically designed for detecting junctions even in genes transcribed at very low levels. Because only 20 % of *S. torvum* reads mapped in eggplant genome with Top Hat, we decide to use a BWA [51], a most suited mapper in the case of greater genetic distance. Subsequently, the reads were realigned using the GATK (Genome Analysis Tool Kit) software in order to split the reads [118].

Raw paired-end reads from *Solanum melongena* and *S. torvum* were downloaded through the NCBI Sequence Read Archive (SRA). The S. melongena reads were deposited under accession number [SRA: SRR1104129] and *S. torvum* reads under accession [SRA: SRR1104128]. The raw sequences were processed and trimmed as described above. The FASTA sequence of the draft eggplant genome was downloaded from the Eggplant Genome Database [119].

### Molecular markers discovery

#### SNVs

SNVs (SNPs and INDELs) were detected using the FreeBayes program [88], a bayesian haplotype-based SNP caller, using the Top Hat alignment. To verify the quality of SNP calling, three SNVs per eggplant chromosome were validated in the sequenced genotypes. Every SNV locus was checked by IGV software [120], to select the most polymorphic loci with the higher coverage. Primers pairs were designed in flanking regions using Primers3 [121].

HRM-based PCR was used to validate the SNPs in a LightCycler 480 Real-Time PCR (Roche, Basel, Switzerland). The reactions were performed in a 10 μL: 5 μL Master Mix 2×, 0.8 μL MgCl2 25 mM, 0.25 μL each primer, 1.7 μL water and 2 μL DNA 30 ng/μL with the following touchdown PCR program: denaturation at 95 °C for 10 min, followed by 55 cycles of 10 s at 95 °C, 15 s at 65 °C (decreasing 1 °C each cycle until 55 °C) and of 15 s at 72 °C, finally the melting at 1 min at 95 °C, 1 min at 40 °C, 1 sec at 60 °C and rising the temperature at 0.02 °C/s until 95 °C.

Although all SNVs matched the quality criteria, not all of them seemed equally reliable. Different filters, developed by ours [113], have been applied to VCF file in order to maximize the polymorphism validation. The settings of the filters are provided in the Additional file 13. The VKS filter was applied to differentiate INDELs from SNPs, the filter CS60 to detect if the SNV was closer than 60 nucleotides to another SNV, the filter CL60 to identify SNVs closer than 60 nucleotides to the transcript edge and the filter HV0.05 to determine if the region had more than 5 SNVs per 100 bases. All these filters allow selecting SVNs, with small amplicons size (e.g. 80-100 bp) and are suitable for manually validation such as with HRM as well as for high-throughput genotyping platform [89, 93, 94]. If the SNV is going to be genotyped by CAPS, the filter CEF will help to select common low-priced digestion enzymes.

#### SSRs

The annotation of SSRs was carried out with Sputnik software [95], selecting the sequences containing ≥ 9 di-, ≥ 6 tri-, or ≥ 4 tetranucleotide motifs. The sequences of unigenes which contain SSRs were blasted against the eggplant genome database in order to know their physical position while their region in the transcripts (ORFs, 3′-UTR and 5′-UTR) were detected using the Bedtools utilities [122]. The representation of the SSRs distribution along the eggplant genome was performed with Circos software. One SSR per eggplant chromosome was selected in order to validate them in the *S. incanum* and *S. aethiopicum* sequenced genotypes.

All selected SSRs for validation were checked via IGV viewer and primers pairs were designed with Primers3. The amplification of SSRs were performed by touchdown PCR in a final volume of 12 μL: 7.21 μL water, 1.2 μL 1× PCR buffer, 0.6 μL MgCl_2_ 50 mM, 0.24 μL dNTPs 10 mM, 0.3 μL reverse primer 10 μM, 0.06 μL forward primer with M13 tail 10 μM, 0.24 μL fluorochrome (FAM, VIC, NED and PET) 10 μM, 0.15 μL Taq DNA Polymerase (5U/μL), 2 μL DNA template 20 ng/μL under the following cycling conditions: denaturation at 95 °C for 3 min, followed by 10 cycles of 30 s at 95 °C, 30 s at 65 °C (with each cycle the annealing temperature decreasing 1 °C), and of 30 s at 72 °C. Products were subsequently amplified for 20 cycles at 95 °C for 30 s, 55 °C for 30 s and 72 °C for 30 s, with a final extension at 72 °C for 5 min.

PCR products were diluted in formamide and analyzed on an automated DNA sequencer ABI PRISM 3100-Avant with a GeneScan 600LIZ (Applied Biosystems, California, USA) size standard. The data were analyzed using the GeneScan software (Applied Biosystems) to obtain the electropherograms and polymorphisms were analyzed with Genotyper DNA Fragment Analysis software (Applied Biosystems, California, USA).

### Ethics and consent to participate

Not Applicable.

### Consent to publish

Not Applicable.

### Availability of data and materials

The datasets supporting the conclusions of this article are available in the Sequence Read Archive at the National Center for Biotechnology Information (NCBI) at the accession number SRS1054263 (http://www.ncbi.nlm.nih.gov/sra?LinkName=biosample_sra&from_uid=4023348) for *S. incanum* and at the accession number SRS1052489 (http://www.ncbi.nlm.nih.gov/sra?LinkName=biosample_sra&from_uid=4025429) for *S. aethiopicum*. The datasets supporting the conclusions of this article are included within the article and its additional files.

## Additional files

Additional file 1:
*Solanum incanum* and *S. aethiopicum* assembled transcripts (compressed folder). The file provides the fasta sequences of the 108,322 *S. incanum* and 106,660 *S. aethiopicum* transcripts. (7Z 38772 kb) Additional file 2: List of *Solanum incanum* and *S. aethiopicum* unigenes (compressed folder). The file provides the list of the 83,905 most expressed single-copy *S. incanum* and 87,084 *S. aethiopicum* transcripts in fasta format. (7Z 29593 kb) Additional file 3:
*Solanum incanum* and *S. aethiopicum* unigene physical positions (compressed folder). Blast search results in order to obtain the *S. incanum* and *S. aethiopicum* unigen positions using *S. melongena* draft genome (cut-off e-value 1e-20). (7Z 22726 kb) Additional file 4:
*S. incanum* and *S. aethiopicum* unigenes potentially encoding proteins (compressed folder). The file contains the unigene protein annotations performed using the three major public and the tomato protein databases (Swiss-Prot, ITAG 2.4, Arabidopsis, Uniref90). (7Z 1181 kb) Additional file 5: GO terms and EC number unigene annotation (compressed folder). The file provides a list of GO terms and EC numbers assigned to *S. incanum* and *S. aethiopicum* unigenes. (7Z 1086 kb) Additional file 6: Biological pathway maps (compressed folder). The file provides the biological pathway maps, obtained by KEGG searches. (7Z 8001 kb) Additional file 7: Details of KEGG annotation (compressed folder). The files provide the names of biological pathways, number of unigenes involved, enzymes types and EC numbers of S. incanum and S. aethiopicum unigenes KEGG annotation. (7Z 56 kb) Additional file 8: Position in tomato reference genome (compressed folder). The file provides the physical position of *S. incanum* and *S. aethiopicum* unigenes in tomato through Blast against *S. lycopersicum* genome, version SL2.5. (7Z 450 kb) Additional file 9:
*Solanum incanum* and *S. aethiopicum* orthologs (compressed folder). The file provides the lists of *S. incanum* and *S. aethiopicum* unigenes which presented orthologs in *S. lycopersicum* obtained through best reciprocal hits using BlastN. (7Z 524 kb) Additional file 10: ORFs and introns annotation (compressed folder). The GFF3 format file provides the ORFs and introns detected in *S. incanum* and *S. aethiopicum* unigenes. (7Z 6710 kb) Additional file 11: SNP calling results. The file in VCF format provides the list of SNVs (SNPs and INDELs) identified in *S. incanum*, *S. aethiopicum*, *S. melongena* and *S. torvum* transcriptomes by mapping against *S. melongena* genome. For each SNV are indicated their position in eggplant genome scaffold, the allele of reference (eggplant genome allele) and the alternative allele (the transcriptomes allele), the quality of the SNV, the filters applied and detailed information about SNP calling process for each SNV and the different kind of transcriptome ID combinations in order to filter the intraspecific and interspecific polymorphisms. The different filter applied for in silico selection were: CS60 (the SNV is closer than 60 nucleotides to another SNV), HV0.05 (the region has more than 5.0 SVNs per 100 bases), CL60 (the SNV is closer than 60 nucleotides to the reference edge), CEF (SNV is not a CAP detectable by the enzyme: cheap_ones), VKF (it is not an SNP). (7Z 38609 kb) Additional file 12: Validated markers. The file provides the tables of SNPs and SSRs experimentally validated. For each marker are provided the unigene which came from, the position in the correspondent eggplant scaffold, the detected alleles and the primers used for validation. (XLSX 21 kb) Additional file 13: Filters settings. The file provides the settings of the filters that have been applied to VCF file in order to filter the SNVs. (TXT 1 kb)

## Abbreviations

EC number: enzyme commission number
ESTs: Expressed sequence tag
FISH: fluorescence in situ hybridization
GATK: genome analysis tool kit
GO term: gene ontology term
HRM: high resolution melting
INDEL: insertion/deletions
KEGG: kyoto encyclopedia of genes and genomes
NCBI: national center for biotechnology information
ncRNAs: noncoding RNAs
NGS: next-generation sequencing
NR database: non-redundant protein database
ORF: open reading frame
SNP: single nucleotide polymorphism
SNVs: single nucleotide variations
SSR: simple sequence repeat
UTR: untranslated region
VCF: variant call format
